# Cancer-Specific Survival after Limb Salvage versus Amputation in Children and Adolescents with Osteosarcoma: A Population-Based Analysis with Propensity Score Matching

**DOI:** 10.1155/2023/8635829

**Published:** 2023-04-08

**Authors:** Zhenwei Li, Bo Xu, Jingjing Cai, Zhengang Zha

**Affiliations:** ^1^Center for Bone, Joint and Sports Medicine, The First Hospital of Jinan University, Jinan University, Guangzhou, China; ^2^Department of Orthopedics, The Second Affiliated Hospital of Bengbu Medical College, Anhui Province, China; ^3^Department of Orthopedic, Shanghai Tenth People's Hospital, Tongji University, Shanghai, China; ^4^School of Clinical Medicine, Bengbu Medical College, Anhui Province, China

## Abstract

**Background:**

The study aims to identify whether osteosarcoma patients of children and young adults will benefit from a survival profit from the choice of the operation method.

**Methods:**

The National Cancer Institute SEER database from 2000 to 2018 was selected for a retrospective analysis of 1630 children and young adults with a primary diagnosis of osteosarcoma, 1222 who underwent limb-preserving surgery, and 408 who underwent amputation. Confounders were controlled for by propensity score matching (PSM), cancer-specific survival (CSS) was analyzed using the Kaplan–Meier method, and univariate and multivariate Cox regression was used to analyze the factors influencing the prognosis of children and young osteosarcoma patients after surgery. A nomogram plot predicted 1-, 3-, and 5- survival rate in osteosarcoma. The model's accuracy was validated by the area under the ROC and calibration curves.

**Results:**

After PSM, multifactor Cox regression analysis found AJCC Stage III-IV (CSS : HR = 5.26, 95% CI 1.95–14.18, *p*=0. 001; HR = 5.54, 95% CI 2.56–12.01, *p* < 0. 001. Limb salvage surgery (CSS : HR = 0.58, 95% CI 0.44–0.77, *p* < 0. 001) has independent impact factors for CSS prognosis. The survival curve before and after PSM showed that patients with osteosarcoma of children and young adults who underwent limb salvage surgery had a survival benefit compared with those who underwent amputation surgery. Gender, chemotherapy, histology, primary tumor site, stage, and surgical modality were modeled in a total of six variables in the nomogram. The model exhibited good predictive performance. The AUC were 0.823, 0.74, and 0.757 for training set at 1, 3, and 5 years, respectively. The AUC of validation set 0.666, 0.722, and 0.699 at 1, 3, and 5 years, respectively. The model also predicted CSS with good fidelity for both datasets. This model was significantly superior to the 8^th^ edition of the AJCC TNM staging system, with a better net benefit in predicting CSS in children and young adults with osteosarcoma.

**Conclusion:**

Limb salvage surgery is an option for children and young adults with osteosarcoma and cancer-specific survival rates can be improved by receiving limb salvage surgery.

## 1. Introduction

Osteosarcoma (OS) is a highly aggressive bone tumor, which typically occurs in the metaphysis of long bones, such as the femur, tibia, and humerus. Children and young adults (CYA) are the most prevalent group with OS, with the peak incidence occurring between the ages 10 and 19 years [1]. OS is caused by a multitude of reasons, including ionizing radiation, alkylation chemicals, chromosomal abnormalities, and hereditary retinoblastoma [2]. The current treatment is that preoperative (neoadjuvant) chemotherapy is used in conjunction with thorough surgical excision, followed by postoperative (adjuvant) chemotherapy [3]. A cornerstone of treatment is a complete surgical excision of original and recurrent or metastatic OS. Amputation used to be the conventional treatment, but it only had a 10–20 percent 5-year survival rate. [4–6]. With advancements in chemotherapy regimens, limb salvage surgery (LSS) has emerged as a feasible option with an enhanced long-term survival rate to 60–80% [7, 8]. However, amputation is required if the tumor at the margin of healthy tissues cannot be removed completely [9]. Similarly, urgent amputation may be indicated when a patient has a serious pathological fracture or chemotherapy is ineffective [10, 11].

Currently, a conflict still persists about the survival outcome between LSS and amputation [12–14]. Furthermore, most earlier studies included patients of various ages, and just a few studies particularly targeted OS patients in CYA. Not only were there no accurate ways for predicting cancer-specific survival (CSS) at the time, but there were also no predictors connected with the survival difference between LSS and amputation. In this study, we developed a predictive model utilizing the National Cancer Institute's Surveillance, Epidemiology, and End Results (SEER) database to evaluate that whether OS patients of CYA will benefit from a survival profit from the choice of operation method.

## 2. Methods

### 2.1. Data Source and Study Population

We retrieved all cases from the SEER database, which is open to the public for research purposes and does not require ethics committee approval or informed consent. The rules of the SEER database are followed in our methodology. We extracted the information from all primary OS patients from 2000 to 2018. Inclusion criteria were as follows: (1) pathological diagnosis of OS; (2) primary sites restricted to extremities and pelvis; (3) known survival status and time; and (4) known primary surgery site codes. Exclusion criteria were as follows: (1) survival time <1 month; (2) unknown operation code; (3) unknown AJCC (American Joint Committee on Cancer) 8th edition stage; (4) unknown survival status, survival time, or cause of death; and (5) age >39.

### 2.2. Data Element

The following data variables were extracted: age, sex, race, primary site, histology (conventional and other), AJCC stage, chemotherapy (no/unknown or yes), and amputation and LSS are two surgical options. CSS was defined as the time from diagnosis to mortality ascribed to OS, which was utilized as the main outcome.

### 2.3. Statistical Analyses

Based on which surgery was performed, the samples were categorized into two groups: LSS group and amputation group. Data were collated. Categorical data were expressed as frequencies or percentages. Chi-square tests were used to identify significant differences between the groups. To balance the number of cases between two groups and reduce the influence of data inaccuracy and confounding variables, we utilized propensity score matching (PSM). The Kaplan–Meier technique (log-rank test) was used to differentiate survival between the LSS group and amputation group.

The eligible patients were randomly divided into a training group and a validation group according to the proportion of 7 : 3. First, to identify risk variables for CSS, researchers employed univariate Cox regression and in order to find independent prognostic factors, variables with *p* < 0.05 in univariate analysis were included in the multivariate Cox regression analysis. Subsequently, CSS nomograms were created based on independent risk variables. The C-index and ROC curve were used to evaluate the discrimination ability of the nomogram. The calibration plot was used to evaluate the accuracy of the nomogram. According to the optimal critical value of the clinical predictive model, the patients were divided into a low-risk group and a high-risk group, and the prognostic value of nomogram was analyzed by Kaplan–Meier technique (log-rank test). All results were expressed as hazard ratios (HR) and their respective 95% confidence intervals (CI), and *p* < 0.05 was considered statistically significant.

Statistical analyses were performed using SPSS (version 24.0, IBM, USA), *R* (version 4.0.3, University of Auckland, New Zealand), and X-TILE software (Yale University in the United States).

## 3. Results

### 3.1. Patient Characteristics

**Figure 1:**
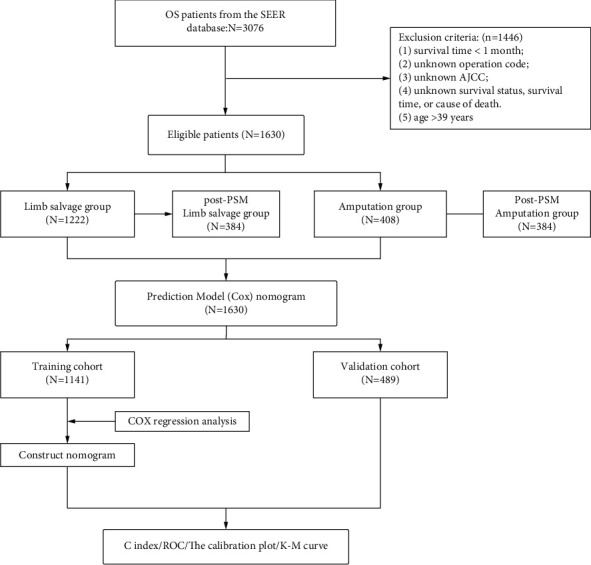
Flowchart.

**Figure 2:**
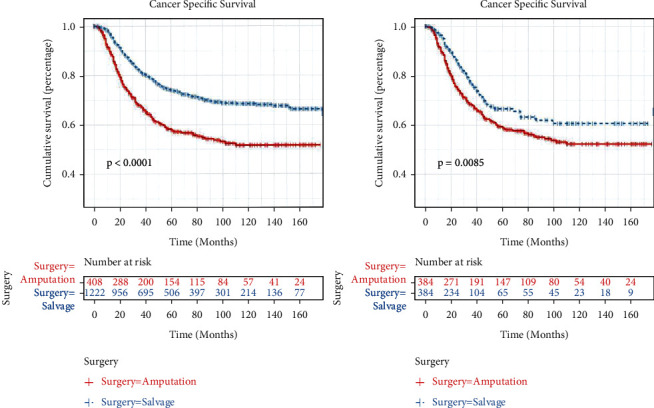
Survival curves of the LSS and amputation in the OS original and matched cohorts. (a) Original cohort and (b) matched cohort.

Our study cohort included a total of 1630 OS patients under the age of 39 years between 2000 and 2018 obtained from the SEER database. The LSS group included 1222 (75.0%) patients and the amputation group included 408 (25.0%) patients (Figure 1). Compared with the amputation group, the LSS group had more female, more lower of primary sites, less conventional of histology, and more AJCC stage II (*p* < 0.05). After the 1 : 1 PSM, the survival analysis included 384 patients, each of who underwent amputation or LSS. Moreover, in the post-PSM data, the baseline characteristics—gender, primary tumor site, histology, and AJCC stage—were all balanced (*p* > 0.05) (Table 1). According to the K–M analysis and log-rank test, the LSS group had longer CSS than the amputation group in the original and matched cohorts (*p* < 0.01) (Figure 2).

### 3.2. Independent Prognostic Factors for OS

Univariate Cox regression analysis was performed on the original cohort first, and variables with *p* < 0.05 were included in the multivariate Cox regression analysis. It was found that LSS and histologically unconventional treatment were protective factors, and male and pelvic in the primary site and AJCC stage III and IV were risk factors (Table 2). In the same way, in the matched cohort after PSM, LSS was a protective factor and AJCC stage III and IV were risk factors. It indicated that LSS was a stable protective factor for CSS (Table 3).

### 3.3. Nomogram Construction and Validation

**Figure 3:**
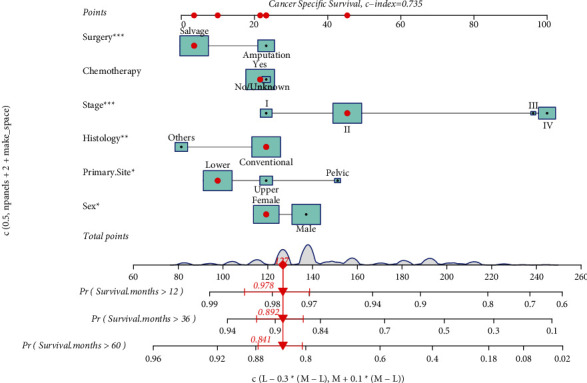
Nomograms that forecast 1-, 3-, and 5-year CSS. The total points were calculated by summing the points from each predictor and correlate to the chances of 1-, 3-, and 5-year CSS in osteosarcoma patients.

**Figure 4:**
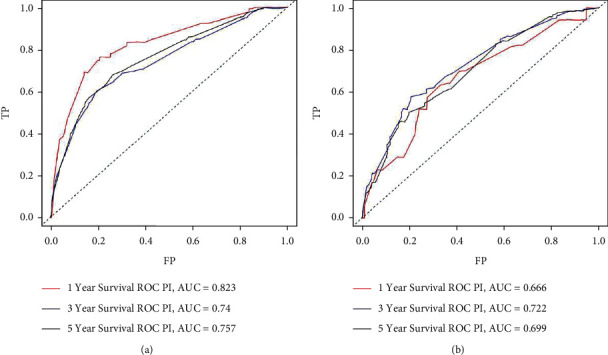
ROC curves for predicting 1-, 3-, and 5- years OS in the training and validation cohorts. (a) Training cohort and (b) validation cohort.

**Figure 5:**
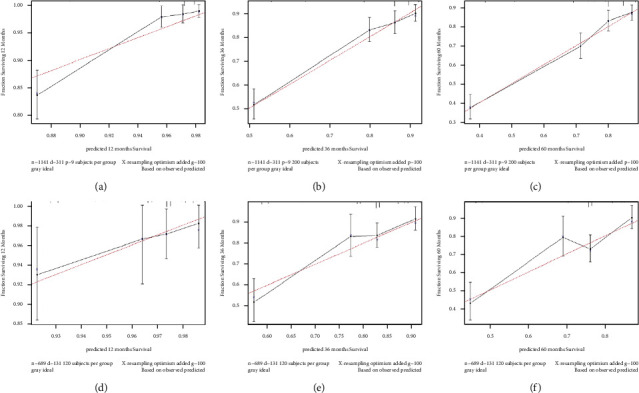
Calibration curves for 1-, 3-, and 5-year OS of the training and validation cohort. (a) Calibration curve for 1-year OS of the training cohort, (b) calibration curve for 3-year OS of the training cohort, (c) calibration curve for 5-year OS of the training cohort, (d) calibration curve for 1-year OS of the validation cohort, (e) calibration curve for 3-year OS of the validation cohort, and (f) calibration curve for 5-year OS of the validation cohort.

CSS nomograms were developed based on the findings of multivariable Cox regression studies (Figure 3). Using these nomograms, the 1-, 3-, and 5- years survival probability of each patient can be predicted by adding up the specific numerical value of each predictive variable. The C-index of the established nomograms showed good predictive accuracy in CSS (C-index 0.735). The area under curve (AUC) for CSS at 1-, 3-, and 5-years in the training set was 0.823, 0.740, and 0.757, respectively, according to the ROC curve analysis (Figure 4(a)). The area under the curve (AUC) for CSS at 1-, 3-, and 5-years in the validation set was 0.666, 0.722, and 0.699, respectively, as a contrast (Figure 4(b)). The calibration curves of CSS show that there was a good agreement between the predicted survival probability and the observed survival probability on the training set and the validation set (Figure 5(a)–5(f)).

### 3.4. Risk Classification Systems for CSS

**Figure 6:**
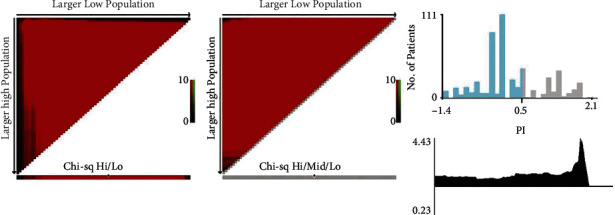
The optimal cutoff value of the score of the prognostic score.

**Figure 7:**
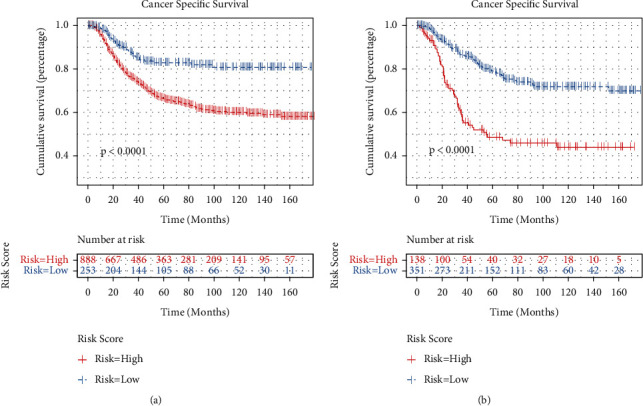
Survival curves of the low- and high-risk groups in the OS in the training cohort and the validation cohort: (a) training cohort and (b) validation cohort.

Each patient's prognosis score was determined by adding the individual results for each component. The patients were then divided into low- and high-risk groups based on the optimal cutoff value of the score as computed by the X-TILE program. The optimal cutoff value is 0.5 (Figure 6). The Kaplan–Meier curves showed a significant difference in training cohort and validation cohort between the two groups (*p* < 0.05) (Figure 7(a) and 7(b)).

## 4. Discussion

The multivariable logistic analysis indicated that male and pelvic in the primary site were independent risk factors for OS patients of CYA, which was consistent within a previous study [15].Males have been found to have a faster rate of bone growth than females [16]. Numerous studies have reported that as the height of an individual increases so does the risk of OS [17–19]. Pelvic osteosarcoma has a substantially worse prognosis than osteosarcoma of the extremities [20]. This study also confirmed the AJCC stage was an independent risk factor that affected the prognosis of OS patients. AJCC stage III and IV usually means that OS invaded the surrounding critical blood vessels and nerves around it, and even has distant metastases. At present, the fraction of cancer that is lowly differentiated or undifferentiated is usually larger, and this is thought to be the leading cause of death in OS [21].

In this study, 75.0 percent of patients (1222/1630) received LSS, which was comparable to previous studies [22, 23]. However, compared with previous studies, we focused on OS patients of CYA. The number of patients treated with LSS has been growing, owing mostly to the development of the multidisciplinary therapy and maturity of prosthesis technology [24]. 3D printing prosthesis has been used to treat malignant bone tumors28, using a combination of surgical reconstruction technology and computer science and technology. Surgical reconstruction can provide tailored treatment for patients of this age. [25]. Patients at the age can achieve individualized treatment by surgical reconstruction. Consequently, they may conquer their fear, overcome their inferiority complex, regain their confidence, lessen the load on their families, and improve the LSS rate.

Several previous investigations have found similar results [26, 27]. In this study, LSS was found to have a higher CSS rate than amputation. This is most likely due to the intrinsic selection bias of amputation for more aggressive OS that do not react well to chemotherapy, include critical neurovascular structures, or are so massive that the patient would be left with a nonfunctional limb following LSS. Although current surgery tends to be LSS, few studies have specifically addressed OS patients of CYA. In addition, previous studies have been small sample sizes and single-center studies and the conclusions might be impacted by confounding variables. Therefore, PSM was utilized in the current study to help control for some possible confounders, specifically the propensity to receive certain therapies based on tumor and patient characteristics.

There is a crucial outcome that we must keep an eye on. We found that LSS was a stable protective factor for CSS. Considering the significant survival benefits on OS such as preserving limb function, improving the quality of life, and eliminating psychological and social obstacles, LSS should be recommended as the optimal surgical procedure for patients of CYA with OS if they were eligible for this surgical procedure. Carefully, preoperative MRI should be used to assess the connection between the tumor edge and the epiphysis plate.

In the present study, we constructed a nomogram to predict 1 -, 3-, and 5-year CSS for OS patients of CYA and internal verification was carried out. C-index, AUC, and the calibration curve showed that the model indicated good performance of predicting 1-year, 3-year, and 5-year CSS in OS patients at the age. There is currently no comparable nomogram model for CYA with OS. According to the nomogram model we developed, the survival probability of an individual OS patient may be predicted more precisely. Doctors may identify patients and make personalized treatment based on the nomogram in order to enhance therapy effectiveness and patient prognosis.

It is worth mentioning that this study has several limitations. First, the SEER database lacked poor treatment information and did not give specifics such as particular chemotherapy, targeted therapy, and immunotherapy, which limited the precise examination of the therapeutic effect on OS. Second, because the codes for particular surgical procedures such as local excision, partial resection, and radical excision varied over time in the SEER database, a subgroup analysis of specific surgical procedures such as LSS and amputation was not undertaken. Third, the SEER database did not collect information about local recurrence and metastasis, so it could only take patients' 1-, 3-, and 5-year survival rates as the main end point. Due to the retrospective study design based on the SEER database, we were unable to draw a firm conclusion on the causal relationship between various variables and surgical choice.

## 5. Conclusion

To summarize, we established and validated a novel nomogram for OS patients of CYA, which could serve as concise and practical tools for clinicians to anticipate the 1-, 3-, and 5- years CSS. LSS for patients with OS exhibited significant benefit on CSS compared with amputation. While new chemotherapy regimens will be required to increase survivorship in the setting of OS, patients with tumor features suitable to LSS had a much higher survival rate than those suitable for amputation. [28–30].

## Figures and Tables

**Table 1:** Demographic data and baseline characteristics of OS patients of CYA in original and matched cohorts.

	*The original cohort*				*The matched cohort*			
	LSS group (*N* = 1222)	Amputation group (*N* = 408)	*P* value	Overall (*N* = 1630)	LSS group (*N* = 384)	Amputation group (*N* = 384)	*P* value	Overall (*N* = 768)
*Age*			0.954				0.83	
<15	531 (43.5%)	176 (43.1%)		707 (43.4%)	166 (43.2)	162 (42.2)		328 (42.7%)
15–39	691 (56.5%)	232 (56.9%)		923 (56.6%)	218 (56.8)	222 (57.8)		440 (57.3%)

*Sex*			<0.001				0.94	
Female	557 (45.6%)	156 (38.2%)		713 (43.7%)	143 (37.2)	145 (37.8)		288 (37.5%)
Male	665 (54.4%)	252 (61.8%)		917 (56.3%)	241 (62.8)	239 (62.2)		480 (62.5%)

*Race*			0.341				0.85	
White	908 (74.3%)	306 (75.0%)		1214 (74.5%)	295 (76.8)	298 (77.6)		593 (77.2%)
Black	189 (15.5%)	53 (13.0%)		242 (14.8%)	39 (10.2)	41 (10.7)		80 (10.4%)
Others	125 (10.2%)	49 (12.0%)		174 (10.7%)	50 (13.0)	45 (11.7)		95 (12.4%)

*Primary site*			<0.001				0.68	
Upper	193 (15.8%)	62 (15.2%)		255 (15.6%)	69 (18.0)	60 (15.6)		129 (16.8%)
Lower	999 (81.8%)	314 (77.0%)		1313 (80.6%)	299 (77.9)	308 (80.2)		607 (79.0%)
Pelvic	30 (2.5%)	32 (7.8%)		62 (3.8%)	16 (4.2)	16 (4.2)		32 (4.2%)

*Histology*			0.045				1.00	
Conventional	1008 (82.5%)	354 (86.8%)		1362 (83.6%)	334 (87.0)	335 (87.2)		
Others	214 (17.5%)	54 (13.2%)		268 (16.4%)	50 (13.0)	49 (12.8)		99 (12.9%)

*AJCC stage*			<0.001				0.99	
I	154 (12.6%)	37 (9.1%)		191 (11.7%)	33 (8.6)	34 (8.9)		67 (8.7%)
II	809 (66.2%)	233 (57.1%)		1042 (63.9%)	229 (59.6)	224 (58.3)		453 (59.0%)
III	23 (1.9%)	13 (3.2%)		36 (2.2%)	8 (2.1)	8 (2.1)		16 (2.1%)
IV	236 (19.3%)	125 (30.6%)		361 (22.1%)	114 (29.7)	118 (30.7)		232 (30.2%)

*Chemotherapy*			0.115				0.52	
No/unknown	106 (8.7%)	25 (6.1%)		131 (8.0%)	18 (4.7)	23 (6.0)		41 (5.3%)
Yes	1116 (91.3%)	383 (93.9%)		1499 (92.0%)	366 (95.3)	361 (94.0)		727 (94.7%)

**Table 2:** Univariate and multivariate Cox regression analysis for OS in the original cohort.

Characteristics	*The univariate analysis*			*The multivariate analysis*		
	HR	CI.95	*P* value	HR	CI.95	*P* value
*Age*						
<15	Reference					
15–39	1.2	0.99–1.45	0.059			

*Chemotherapy*						
No/Unknown	Reference			Reference		
Yes	2.77	1.66–4.64	<0.001	1.2	0.65–2.2	0.5597

*Histology*						
Conventional	Reference			Reference		
Others	0.45	0.31–0.59	<0.001	0.57	0.4–0.79	0.001

*Primary site*						
Upper	Reference			Reference		
Lower	0.83	0.64–1.06	0.135	0.85	0.66–1.1	0.2148
Pelvic	1.94	1.27–2.97	0.002	1.91	1.24–2.95	0.0035

*Race*						
White	Reference					
Black	1.21	0.94–1.56	0.135			
Others	1.15	0.85–1.55	0.367			

*Sex*						
Female	Reference			Reference		
Male	1.28	1.06–1.55	0.01	1.22	1.01–1.48	0.04

*Stage*						
I	Reference			Reference		
II	2.46	1.43–4.23	0.001	1.71	0.91–3.23	0.0951
III	9.04	4.59–17.77	<0.001	6.5	3.09–13.68	<0.001
IV	9.07	5.27–15.61	<0.001	6.05	3.19–11.44	<0.001

*Surgery*						
Amputation	Reference			Reference		
LSS	0.54	0.45–0.66	<0.001	0.66	0.54–0.8	<0.001

**Table 3:** Univariate and multivariate Cox regression analysis for OS in the matched cohort.

Characteristics	*The univariate*			*The multivariate*		
	HR	CI.x	*P* value	HR.y	CI.y	*P* value
*Age*						
<15	Reference					
15–39	1.25	0.95–1.64	0.111			

*Chemotherapy*						
No/unknown	Reference					
Yes	1.52	0.78–2.96	0.219			

*Histology*						
Conventional	Reference			Reference		
Others	0.58	0.37–0.92	0.020	0.63	0.4–1.01	0.055

*Primary site*						
Upper	Reference					
Lower	0.73	0.52–1.02	0.063			
Pelvic	1.55	0.88–2.75	0.132			

*Race*						
White	Reference			Reference		
Black	1.47	1.01–2.13	0.045	1.22	0.84–1.78	0.302
Others	0.93	0.61–1.42	0.748	1.06	0.69–1.61	0.799

*Sex*						
Female	Reference					
Male	1.14	0.86–1.5	0.368			

*Stage*						
I	Reference			Reference		
II	1.52	0.7–3.28	0.291	1.27	0.58–2.78	0.5511
III	5.78	2.15–15.51	0.001	5.26	1.95–14.18	0.001
IV	6.23	2.91–13.33	<0.001	5.54	2.56–12.01	<0.001

*Surgery*						
Amputation	Reference			Reference		
LSS	0.69	0.52–0.91	0.009	0.58	0.44–0.77	<0.001

## Data Availability

Publicly available datasets were used in this study. These data can be found in the Surveillance Epidemiology and End Results (SEER) database.
